# Exploring stakeholders’ perceptions of a task-shifting strategy for hypertension control in Ghana: a qualitative study

**DOI:** 10.1186/s12889-017-4127-9

**Published:** 2017-02-21

**Authors:** Juliet Iwelunmor, Joyce Gyamfi, Jacob Plange-Rhule, Sarah Blackstone, Nana Kofi Quakyi, Michael Ntim, Ferdinand Zizi, Kwasi Yeboah-Awudzi, Alexis Nang-Belfubah, Gbenga Ogedegbe

**Affiliations:** 1 Department of Kinesiology and Community Health, University of Illinois Urbana-Champaign, College of Applied Health Sciences, Champaign, USA; 2 Department of Population Health, New York University School of Medicine, New York, USA; 3 Kwame Nkrumah University of Science and Technology, Kumasi, Ghana; 4 Ghana Health Services, Accra, Ghana

## Abstract

**Background:**

The purpose of this study was to explore stakeholders' perception of an on-going evidence-based task-shifting strategy for hypertension (TASSH) in 32 community health centers and district hospitals in Ghana.

**Methods:**

Using focus group discussions and in-depth interviews, qualitative data were obtained from 81 key stakeholders including patients, nurses, and site directors of participating community health centers involved in the TASSH trial. Qualitative data were analyzed using open and axial coding techniques.

**Results:**

Analysis of the qualitative data revealed three themes that illustrate stakeholders' perceptions of the ongoing task-shifting strategy for blood pressure control in Ghana and they include: 1) awareness and understanding of the TASSH program; 2) reasons for participation and non-participation in TASSH; and 3) the benefit and drawbacks to the TASSH program.

**Conclusion:**

The findings support evidence that successful implementation of any task-shifting strategy must focus not only on individual patient characteristics, but also consider the role contextual factors such as organizational and leadership factors play. The findings also demonstrate the importance of understanding stakeholder's perceptions of evidence-based task-shifting interventions for hypertension control as it may ultimately influence the sustainable uptake of these interventions into "real world" settings.

## Background

Stakeholders’ perceptions of the implementation of evidence-based task-shifting strategies in low- and middle - income countries (LMICs) including Ghana are important for several reasons. First, decisions regarding transferring research to practice are often complex, involving a number of policymakers and other key stakeholders [1–3]. There is a need to support these decisions with the best evidence available, particularly evidence that takes the perceptions of stakeholders into consideration [4, 5]. Second, it can take many years for a new intervention to be broadly implemented [6], and implementation and scale-up may involve multiple stakeholders operating in a nexus of differing agendas, priorities, leadership styles, and negotiation strategies [7]. In these circumstances, the perceptions of key stakeholders are needed to clearly articulate who answers to whom, what communication methods are to be utilized, and how to best facilitate a sense of inclusion and support for \ implementation from the beginning [7]. To date however, and as illustrated by a recent systematic review, little is known about stakeholders’ perceptions of the implementation of evidence-based task-shifting strategies for blood pressure (BP) control in LMICs [8]. As stakeholders have the ultimate say as to what evidence is adopted and used, understanding their perceptions may guide efforts to scale-up known evidence-based task-shifting interventions suitable for low resource setting in Sub-Saharan Africa (SSA) [6].

Task-shifting strategy is defined as the rational movement of primary care duties from physicians to non-physician health care workers, such as nurses, pharmacists, or community health workers [9, 10]. In sub-Saharan Africa, where there are limited health care resources, task-shifting is a feasible method of implementing primary and secondary prevention at the patient level [11]. Previous studies have indicated that task-shifting is an effective and feasible method in sub-Saharan Africa for addressing hypertension [12–14], however none of these previous studies have taken place in Ghana where hypertension is the second leading cause of outpatient morbidity and mortality [15].

In 2012, a cluster randomized task-shifting strategy for hypertension (TASSH) control trial was implemented in 32 community health centers (CHCs) and district hospitals in Ghana [16]. This study evaluates among hypertensive patients who receive care in CHCs, the comparative effectiveness of the implementation of the World Health Organization Package of Essential Non-Communicable Disease Intervention for Primary Care (WHO PEN) program targeted at cardiovascular risk assessment and hypertension control (intervention group), versus provision of health insurance coverage (control group), on BP reduction at 12-months and sustainability at 24-months [16]. The findings will provide needed information with respect to comprehensive cardiovascular risk reduction and hypertension control in Ghana. However, given that the scaling-up of complex health interventions to large populations is not a straightforward task [6, 17], there is a need to understand stakeholders’ own unique perceptions of the implementation of TASSH.

The purpose of this study is to explore stakeholders’ (i.e., patients, trained TASSH nurses, site directors of participating community health centers and district hospitals) perceptions of the on-going task-shifting cluster-randomized control trial for hypertension in Ghana. The valuable and often understudied perspectives of stakeholders in LMICs may provide necessary insight to inform rapid scale-up of known evidence-based interventions implemented in settings like Ghana.

## Methods

### The TASSH Intervention

TASSH is a 5-year cluster-randomized trial currently in its fifth year and designed to evaluate the comparative effectiveness of the implementation of the WHO-PEN [18] targeted at cardiovascular risk assessment (intervention group) versus provision of health insurance coverage (control group) on blood pressure reduction at 12 months and blood pressure control at 24 months post-intervention. A full description of the TASSH protocol has been published elsewhere [16], but briefly, a total of 32 CHCs and district hospitals in the Ashanti Region of Ghana, were randomly assigned to either the intervention group (*n* = 16) or the control group (*n* = 16). Both patients in the intervention and control group received health insurance and follow-up care every three months. However, patients in the intervention condition received care from community-health nurses who had been trained in blood pressure measurement, hypertension diagnosis, treatment and management, in addition to the WHO Package of Essential Noncommunicable diseases (WHO-PEN) Patients in the intervention group received “usual care,” which varied based on the clinic they attended. Blood pressure outcomes are evaluated at baseline, 12 months, and 24 months. To date, the trial has enrolled 757 patients, of which 86% of all the patients have completed the primary outcome of the study at 12 months. A total of 64 nurses (2 from each of the 32 study sites) were trained in the delivery of the TASSH protocol. Nurses were recruited from each of the 32 clinics involved in the study, and trained in theWHO-PEN CVD Package. The WHO-PEN involved fours steps: 1) inquiry about the patient’s history (e.g., heart attack, stroke, lifestyle behaviors, diabetes); 2) physical and laboratory experiments (including BP measurements, fasting glucose, cholesterol); 3) estimation of cardiovascular disease risk based on risk charts provided by WHO (categorized as low, medium, or high); 4) initiation of drug therapy, lifestyle counseling, and follow-up visits [19, 20]. Nurses working at the intervention clinics implemented these protocols immediately after training, while those working in the control clinics delivered “care as usual” until the end of the intervention, when they provided a delayed intervention, implementing the WHO-PEN training. Treatment received by patients attending the control clinics varied based on clinic procedures; however patients were treated by physicians and not community health nurses. Prior to implementing the TASSH program, nurses’ roles in the clinics included checking and recording vital signs, dressing wounds, taking blood samples to the lab, making beds, dusting or general upkeep of wards, etc.. In the control clinics, nurses maintained these duties until the end of the intervention period, in which they began to implement TASSH.

### Data collection

Data for this present study were obtained from focus group discussions and semi-structured interviews with key stakeholders including patients, nurses, and site directors of participating community health centers involved in the TASSH trial. We used purposive sampling methods [21] (that is, we intentionally sought to interview participants with certain characteristics related to the TASSH trial including randomization group assignment, rural/urban, etc.) to ensure a range of demographic variables and experiences. Written and verbal consent was obtained from all study participants. Each focus group and interview was conducted in Twi or English and lasted approximately 60-90 min. Five focus group discussions were held separately for the patients, trained nurses and the site directors. The focus groups were conducted by TASSH research coordinators fluent in both English and Twi. A semi-structured guide with open-ended questions was used for all discussions. This approach allowed interviewers to tailor questions and probes as needed for the different participants. The open-ended questions also allowed participants to elaborate on issues they consider important or relevant. Sample questions include: Can you describe your personal experience with the TASSH intervention? What information or support did you receive as part of the TASSH program? Was it helpful or unhelpful? Why? Interviews were audio taped, transcribed in Twi, and translated in to English by two bilingual Twi-English study team members. Transcripts were reviewed for accuracy, and the final English transcripts were used for the data analysis process. Data were collected in each stakeholder group until saturation was reached, or no new themes emerged. The study was approved by the institutional review boards of the University of Illinois Urbana-Champaign, New York University School of Medicine and the Committee for Human Research, Publications and Ethics at Kwame Nkrumah University of Science and Technology, Kumasi, Ghana.

### Data analysis

We analyzed the data using an iterative process. The statements from the brainstorming exercises and transcripts from focus group discussions and in-depth interviews were transcribed verbatim and exported into Microsoft Excel. Two of the authors independently reviewed the data. The data was analyzed using an inductive approach, without the presupposition of an existing theoretical framework. According to Patton [22], it is important to conduct this phase of analysis without the presupposition of a particular framework to allow flexibility in data exploration and discovery. Using coding techniques (such as open and axial coding) as described by Strauss and Corbin [23, 24], the researchers open coded the transcripts. Open coding entailed holistically reviewing the data, reading line by line, reviewing each individual response, comparing and cross-checking the responses of participants to the same questions, labeling concepts, and breaking data down into categories that best fit the research questions. Axial coding involved exploring the data (i.e., open codes) for connections between categories and sub-categories. Two of the authors (JI and JG) met after coding the data to discuss the coding process, and discrepancies in coding were discussed until consensus was reached. Quotations were used from the data to illustrate each important theme identified. Credibility and internal validity of the data were assured via use of multiple data sources (focus groups and individual interviews) that allowed for triangulation of data [25]. We also used member checking during and after data collection to verify the information collected and interpretation of our findings. Member checking refers to returning to the participants to ensure that the researchers’ interpretations aligned with participants’ intended messages [25].

## Results

A total of 81 stakeholders (42 patients, 27 nurses, and 12 site directors, which included 4 physicians) participated in this study. Among the patients, 14 were women, 7 patients had no formal education, 11 had elementary education, while the rest had high school education and beyond. The mean age of the patients was 62 years. Among the nurses, 21 were women, mostly young with a mean age of 28 years. Among the site directors, the majority (8) were men with a mean age of 39 years.

Three over-riding themes illustrating stakeholders’ perceptions of the task-shifting strategy for blood pressure control emerged from the interviews: 1) awareness and understanding of the TASSH program; 2) reasons for participation and non-participation in TASSH; and 3) benefit and drawbacks to the TASSH program.

### Patients

#### Awareness and understanding of the TASSH program

Most patients reported first hearing about the TASSH program from their physicians and nurses at the CHCs or district hospital they visited. Others shared that they became aware of the program through announcements in church or when TASSH nurses visited the local marketplace to recruit participants. One patient shared her experience with becoming aware of the TASSH program with the following:

*“I heard of TASSH at the marketplace when the nurses came around. After my blood pressure was checked and I saw it was high, I felt the need to join the program.”*

In describing their understanding of the TASSH programs, participants shared that they enjoyed their interactions with the TASSH nurses and welcomed the opportunity to meet with them, especially since the nurses provided necessary information on ways to control their blood pressure.

One patient shared the following:

*“My blood pressure is better now compared to the pre-TASSH era. I enjoy the constant contact between us the patients and the TASSH nurses. Their education on lifestyle changes, especially on diet has helped me.”*

#### Reasons for participation and non-participation in TASSH

Patients’ participation in TASSH was influenced by previous difficulty obtaining care for hypertension. For some, the previous care received for their condition was sporadic and unsatisfactory. One patient stated, *‘I was already hypertensive but was not satisfied with the care given me.”* Also, because most patients could not afford the antihypertensive medications, provision of the medications as part of TASSH program sparked their interest in joining the program. One patient shared the following:

*“I have been told by the doctor that I have hypertension but I couldn’t buy the drugs, until [I joined] this program.”*

Participation was also influenced by the program structure, including education and regular check-ups. One patient stated: *“checking my blood pressure every month, counseling about eating at night and eating fruits are some of the reasons why I like participating in the [TASSH] program.”* For the patients, access to consistent care, medications and education and counseling on lifestyle changes influenced their continued participation in the program.

#### Benefits and drawbacks to the TASSH program

The patients interviewed shared both benefits and drawbacks of participating in the TASSH program. With regards to the benefits, one participant shared the following:

*“I like the program because the prescribed medication regime I am receiving from [the] TASSH* [26] *has less side effects than what was previously provided by my previous doctors at the local clinics.”*

Another participant stated, *“one thing I like about the program is that checking my weight has helped them [the nurses] to become aware of what I am eating.”* Another patient shared the following: *“the educational component, especially the group format is helpful with gaining knowledge about how to manage my blood pressure.”* While some patients felt that the TASSH program provided more individualized treatment and that the nurses were able to offer them support and feedback that they previously had not received, many of them encountered financial challenges with implementing the necessary lifestyle changes. Some patients noted that: *“the lack of money for purchasing healthy food is a problem.”* One patient shared the following statement:

“*The challenge for me with this program is finances, because not all healthy foods you recommended are available in my immediate surroundings, so I need finances to travel a few miles to buy them and they are also expensive.”*

Additionally, participants expressed their concerns with the affordability of the prescribed medications. One participant shared the following:

*“I cannot get the same drugs given to me at TASSH* [26] *in the hospitals. The drugs given to us are very expensive and not covered on NHIS [national health insurance scheme drug formulary]. Drugs will have to be purchased on our own.”*

In summary, patients described that the TASSH program increased their awareness of lifestyle and behavioral changes they could implement to manage their hypertension, and provided education for them to do this. Some patients also described that even though they were aware of their hypertension status prior to TASSH, they were unable to receive necessary care. Thus, prior inability to access proper care was a strong motivator for patients participation in the program. However, lack of finances, limiting patients ability to purchase antihypertensive medication after the TASSH program ended as well as affordability of healthy foods were noted as barriers to hypertension management.

### Nurses

#### Awareness and understanding of the TASSH program

Similar to the patients, almost all the nurses viewed the TASSH program as potentially helping their clients to improve their blood pressure. One nurse shared the following:

*“TASSH has helped many clients with high BP to reduce it from abnormal to normal. It has also helped some patients know more about their condition.”*

#### Reasons for participation and non-participation in TASSH

Nurses’ participated in TASSH because they enjoyed seeing the positive effects of the program and improvements in patient health. Nurses noted that the program was effective in improving lifestyle and healthful behaviors in ways that previous care was not. One nurse shared the following:

“*I have helped them [patients] come to the hospital regularly because they previously just took medication without coming to care for their blood pressure.”*

One nurse described home visits as a critical component of TASSH, particularly for helping patients adherence to their medication:

*“I provide on-on-one interaction with my patient and I have helped them know their blood pressure level and I motivate and encourage them to take their medications. My patients say that it has helped them a lot and they take their medications seriously.”*

The nurses’ involvement in TASSH contrasted to their typical role at their clinics with hypertensive patients, in which previously hypertension diagnosis and management was solely the responsibility of physicians. Nurses were not allowed to treat hypertension.

#### Benefits and drawbacks to the TASSH program

For the nurses, leadership support was described as an important factor with carrying out TASSH duties particularly in cases where the primary duties of the nurses at the clinic were not in alignment with their role as TASSH nurses. Although some nurses expressed that: *“their clinical priorities were in line with TASSH,”* others mentioned that they: *“divide their attention and often do not have enough time to carry out their TASSH duties.”* One nurse however described the support provided by her site director as helpful with the following statement:

*“Even though I have to perform my other duties before TASSH, my site director lets me attend to TASSH patients when they come around, gives me enough time to attend TASSH training, and forwards new patients to me.”*

In addition to leadership support, some of the nurses mentioned that the availability of blood pressure monitors was a potential benefit because *“it [BP equipment] enabled patients to become more knowledgeable about their hypertension,”* and, *“it allowed me to carry out my duties efficiently.”* However, there were drawbacks to having the equipment:

*“The breakdown of the blood pressure machines [is a challenge] because it [BP equipment] is constantly being borrowed and used at the community health centers or district hospitals for non-TASSH programs.”*

Additionally, the logistics involved with implementing the TASSH program was viewed by some nurses with ambivalence. For example, some nurses reported that *“lack of proper [dedicated] space”* at the community health centers for the TASSH program was an important issue that limited their ability to see patients and properly store patient information. In addition to the logistics, some nurses stated that *“the non-availability of drugs, during follow up care”* was a major barrier to implementing TASSH program effectively, particularly as many of the patients cannot afford to buy their medications at the end of the program.

Overall, nurses felt that the TASSH program was helpful in providing awareness of hypertension status and knowledge of hypertension management to patients. Many nurses cited enjoyment of their work in the TASSH program, including providing necessary education and seeing patient improvement, as reasons for participating as providers in the TASSH program. Finally, nurses described that consistent and strong leadership support was one of the most important facilitators to successful implementation of TASSH. Having clinic directors who were knowledgeable about nurses’ duties with TASSH and accommodated their clinic duties and TASSH duties was critical for success of the program. However, several logistical concerns were noted as primary barriers to program success, including lack of space for seeing patients and equipment breakdown.

### Site directors

#### Awareness and understanding of the TASSH program

Majority of site directors viewed TASSH as “*important with helping to address hypertension management in Ghana in general.”* One director described TASSH in the following way: *“TASSH is providing an enabling environment with the skilled personnel to provide better care for hypertension patients.”* Majority of site directors also suggested that training nurses in the management and control of hypertension was appropriate, given the rather high cost associated with management of a condition like hypertension. One director shared the following:

“*The most important thing is training staff as managing NCDs like hypertension is very expensive. Taking blood pressure is a simple activity that our nurses can do and so it is important that we have programs like TASSH to train them.”*

#### Reasons for participation and non-participation in TASSH

In general, some site directors of the CHC and district hospitals did not take a leadership role in TASSH, due to lack of awareness of the program and lack of involvement from the onset. For site directors who were aware of the program, commitment to participate was high at the beginning; however, leadership turnover in some participating sites did not translate into sustained involvement. As a result, for some sites, there was little involvement of the site directors in promoting or engaging with the TASSH intervention. For example, for some directors, our interviews were described as their *“first interactions with the program.”* One site director shared the following:

*“It is serious when a medical coordinator in a facility is not aware that the program is actually in the facility. So, going forward, I think that you need to include the head* [27] *of the facilities right from the beginning. You structure a program or course for them.”*

Another factor influencing non-participation was the lack of information on TASSH study protocols. A site director shared the following:

*“I do not know what to do when a TASSH participant is referred to me, as I am not aware of the study protocols regarding referrals of acute or worsening cases to physicians.”*

These quotations highlight the importance of a top-down approach in clinics participating in the TASSH program to ensure that individuals occupying leadership positons are fully aware, not only of the existence of the TASSH program, but its protocol as well.

#### Benefits and drawbacks to the TASSH program

Among the site directors, the majority expressed their appreciation for the TASSH program, particularly with reducing the workload of physicians. One director shared the following:

*“I find it very difficult to go through all my patient load. So I saw that with the program in place and the nurses handling the patients, there was a marked reduction in my workload, and the nurses spent quality time with the patients, letting them know what they’re supposed to do."*

Another director also shared the following:

*“The shifting of our tasks to the nurses frees up our time and allows us to provide more attentive and holistic care to patients with more acute conditions.”*

The potential benefit of the TASSH program for patients was described by a site director who noted that since implementation of the program, *“more than 75% of the patients in my facility have improved their BP drastically, with some from multi-therapy to mono-therapy [medications], while others are on temporary suspension of their [blood pressure] medication.”* In terms of the potential drawback, staff turnover was a recurring theme shared by most of the site directors (58%). As one director stated the following:

*“Having to train new nurses in the event of transfer of trained nurses to other health centers not involved with the TASSH* [26] *or in the event of maternity leave, may make it difficult to continue the program at participating sites.”*

In summary, the site directors agreed that TASSH plays an important role in promoting awareness of hypertension and chronic disease in general, and in training health care workers to manage such conditions. There were several challenges with regard to site directors’ motivation for participating in TASSH. The primary concern was that several site directors were unaware of the program goals, or in some instances the program itself. This occurred in several instances where there was turnover in clinic administration and replacement staff were not informed of TASSH. Despite this challenge, the site directors noted that a positive benefit of TASSH was that it lightened physician work-loads, allowing them to spend more time with patients with more severe conditions. However, they described that training more nurses may be necessary, as TASSH nurses may transfer to another facility leaving their clinic of origin without a TASSH provider.

## Discussion

In this study, we explored key stakeholders’ perceptions of the implementation of the TASSH program. The findings offer valuable information, as we are only just beginning to understand the role contextual factors play in intervention implementation [28, 29]. Future success will require the participation of the key stakeholders whom interventions would affect. Previous studies [3, 30] also emphasize the need for participatory research during on-going interventions so as to *“produce evidence-based interventions that are more relevant and actionable to policy and practice stakeholders”* [3].

Our findings revealed important insights into the on-going implementation of TASSH, particularly the importance of understanding participant awareness and understanding of a program, reasons for participating or not participating, and the benefits and drawbacks of the program. For example, while it was evident that the TASSH strategy increased patients awareness of their hypertension, with the nurses going above and beyond their call of duty to implement the program, and the site directors expressing satisfaction with the reduction of their patient workload, there were challenges identified by the patients, including the perceived lack of self-efficacy to adopt the recommended lifestyle changes long-term. Similarly, for the nurses, the frequent breakdown of the blood pressure measuring devices as a result of being used by other non-TASSH trained clinic staff was a drawback to the effective implementation of their duties as TASSH nurses alongside lack of leadership support. For the site directors, some did not participate in the TASSH program either due to miscommunication, lack of information on the study protocol or persistent staff turnover. However, directors who were aware of the program, helped nurses fulfill their TASSH duties for example, by relieving them from work to attend TASSH training or referring new TASSH patients to the nurses.

These findings are similar to those reported by Sanjana and colleagues who evaluated task-shifting strategies in the context of HIV counseling and testing services in Zambia [31]. The authors noted that as health facilities become increasingly dependent on non-physician healthcare workers, the issue of long-term maintenance of these strategies must be critically examined, given high turnover rates and staff retention among community health workers.

Our finding that implementation and dissemination of the TASSH program will require continuous engagement of key stakeholders at all levels, including patients, nurses, and administrators, is supported by studies by other investigators. For example, Copper and colleagues [32] as well as Stirman and colleagues [33] reported that sustaining interventions over time will require greater communication to key stakeholders so as to help these programs achieve the ultimate goal of broad public health impact. Chambers et al., also noted that sustaining implementation of task-shifting strategies like the TASSH program will require continuous engagement of key stakeholders throughout the planning, implementation and dissemination processes so as to increase the fit between the intervention and the local context [34]. Our study raises additional questions about the limitations of task-shifting strategies. For example, while the site directors unanimously agreed that training nurses will reduce the workloads of physicians, and thus allow them to address more acute health issues, the site directors indicated their own need for training on the TASSH protocol, as they were not aware of the study protocols regarding referrals of acute or complicated cases to physicians. Moving forward, taking these results into account can have positive implications for task-shifting implementation, as these previous limitations can be addressed. These findings support evidence that successful implementation of any new task-shifting strategy must focus not only on individual patient characteristics, but also consider the role of contextual factors [35, 36].

### Strengths and limitations

A key strength of this study is that it drew upon the perspectives of stakeholders (patients, nurses, site directors) involved in an on-going task-shifting strategy for blood pressure control in Ghana. In doing so, it has been possible to identify similar and sometimes contradictory perceptions of the TASSH trial. On the other hand, there are limitations in the current study worth mentioning. First, we only interviewed a small sample of stakeholders who may not be representative of all the stakeholders involved with the TASSH trial. It is also likely that our sampling strategy introduced a positive bias in that most of the participants in the study were more likely to be positively predisposed to the TASSH program. As a result, it is possible that participants not interviewed may have views that were not reflected in these findings. To counteract this bias, we did interview site directors and staff with less or no direct involvement in the TASSH trial to explore their unique perspectives on the program. Additionally, the focus discussions and interviews likely introduced a social desirability bias as participants may have responded in a way they perceived as being socially acceptable. That said, the nature of qualitative inquiry is to understand the depth of otherwise unknown information / knowledge such as stakeholders’ perceptions of implementation of interventions in LMICs, and to a certain extent, the aforementioned limitation cannot be avoided given this type of inquiry. Despite these limitations, the knowledge derived from our study has the potential to inform the transfer of research to practice by providing key stakeholders with the best evidence available on the benefits as well as drawbacks associated with implementing interventions in LMICs.

### Implications for evidence-based implementations in low-and middle- income countries

The findings from the current study have several implications not only for researchers and practitioners that implement interventions in LMICs, but also for the funders and technical assistance providers that support them. First, our findings highlight the need for on-going evaluations of task-shifting strategies to explore key stakeholders’ perceptions, which can provide insight for understanding stakeholders’ awareness of the program, reasons for participation or nonparticipation, as well as perceived benefits and drawbacks. Second, the results of this study show that different stakeholders have different viewpoints, for example, of what are the benefits or drawbacks of evidence-based implementations. Hence, what may be considered important for some stakeholders (i.e., nurses), may be different for others (i.e., patients, or site directors). Given the importance and cost of implementing programs like TASSH, further research to understand how individual interests or needs of stakeholders translate into decision-making at the policy level are needed [1, 4]. Finally, as the societal costs of cardiovascular disease prevention are enormous for many LMICs [37–41], sustainability of interventions for CVD prevention remains a critical issue. Addressing this issue will require, among other factors, greater communication with key stakeholders to identify solutions that maximize intended benefits for service provision (i.e., task-shifting) and ultimately care to patients.

## Conclusion

Overall this study provides important contributions to the literature about stakeholders’ perceptions of the implementation of task-shifting strategies for hypertension control. By providing insights into perspectives of key stakeholders, this study offers evidence on potential factors likely to influence rapid scale-up and dissemination of evidence-based CVD prevention services in SSA. These findings have implications for the successful implementation of task-shifting strategies as they demonstrate the importance of understanding participant awareness and understanding of a program, reasons for participating or not participating, and the benefits and drawbacks of the program. The findings may also be used as the basis for the development of more theoretically-informed research that seeks to ensure that interventions for hypertension control are taken up in practice, ultimately achieving results at full scale for CVD improvement efforts in Ghana and other LMICs.

## Abbreviations

CHCs: Community Health Centers
GACD: Global Alliance for Chronic Diseases
SSA: Sub-Saharan Africa
TASSH: Task-Shifting Strategy for Hypertension
WHO-PEN: World Health Organization Package of Essential Non-Communicable Disease Intervention for Primary Care
